# Management strategies and clinical outcomes in breast cancer patients who develop left ventricular dysfunction during trastuzumab therapy

**DOI:** 10.1186/s40959-021-00099-7

**Published:** 2021-03-26

**Authors:** Ren Jie Robert Yao, Jordan Gibson, Christine Simmons, Margot K. Davis

**Affiliations:** 1 Department of Medicine, University of British Columbia, Vancouver, BC Canada; 2 Department of Medicine, University of Calgary, Calgary, AB Canada; 3 Division of Medical Oncology, BC Cancer Agency, Vancouver, BC Canada; 4 Division of Cardiology, University of British Columbia, Vancouver, BC Canada; 5 Gordon & Leslie Diamond Health Care Centre, 2775 Laurel St., 9th Floor, Vancouver, BC V5Z 1 M9 Canada

**Keywords:** Trastuzumab, Left ventricular dysfunction, Heart failure, Breast cancer

## Abstract

**Background:**

Trastuzumab reduces risk of breast cancer recurrence but carries risk of cardiotoxicity that may be reversible upon treatment cessation and institution of left ventricular (LV) enhancement therapies (LVETx). We assessed management patterns of trastuzumab-induced cardiotoxicity (TIC) in a contemporary real-world setting.

**Methods:**

We reviewed charts of all breast cancer patients who received adjuvant trastuzumab in British Columbia between January 2010 and December 2013, spanning the opening of a cardio-oncology clinic. LV dysfunction (LVD) was classified as minimal (LVEF nadir 45–49%), mild (40–44%) or moderate-severe (< 40%). Charts were reviewed for baseline characteristics, management strategies, and outcomes. Multivariable analysis was performed to identify patient characteristics associated with trastuzumab completion and cardiology referral.

**Results:**

Of 967 patients receiving trastuzumab, 171 (17.7%) developed LVD, including 114 patients (11.8%) with LVEF declines of ≥10 to < 50%. Proportions of patients receiving cardiology referrals and LVETx increased and wait times to consultation decreased after a dedicated cardio-oncology clinic opened. LVETx was used more frequently in patients with moderate-severe LVD compared to minimal or mild LVD. Factors associated with completion of trastuzumab included mastectomy (OR 5.1, 95% CI 1.1–23.0) and proximity to quaternary care centre (OR 7.7, 95% CI 2.2–26.2). Moderate-severe LVD was associated with a lower probability of completing trastuzumab (OR 0.07 vs. minimal LVD, 95% CI 0.01–0.74). Factors associated with cardiology referral included heart failure symptoms (OR 8.0, 95% CI 1.5–42.9), proximity to quaternary care centre (OR 6.8, 95% CI 1.3–34.2), later year of cancer diagnosis (OR 2.4 per year, 95% CI 1.4–4.3), node-positive disease (OR 0.18, 95% CI 0.06–0.56), mastectomy (OR 0.05, 95% CI 0.01–0.52), and minimal LVD (OR 0.14, 95% CI 0.05–0.46). LVEF recovered to > 50% in 90.7% of patients.

**Conclusions:**

Management strategies in patients with TIC are associated with cancer characteristics and severity of cardiotoxicity. Access to dedicated cardio-oncology clinics may facilitate optimal care of this complex patient population.

## Introduction

Trastuzumab is a recombinant humanized monoclonal antibody directed against the human epidermal growth factor receptor-2 (HER2) receptor, which is overexpressed in 20–30% of breast cancers and associated with aggressive tumour activity [1]. Trastuzumab induces cell death through antibody-dependent cellular cytotoxicity in cells overexpressing HER2, and has revolutionized the care of breast cancer patients by demonstrated marked survival benefit in adjuvant and metastatic settings [2, 3]. In landmark trials of trastuzumab in combination with chemotherapy in operable breast cancer, this agent was associated with a 50% reduction in risk of recurrence and a 33% reduction in the risk of death [4].

While trastuzumab improves breast cancer outcomes, it is also associated with a risk of trastuzumab-induced cardiotoxicity (TIC). The landmark HERA trial of adjuvant trastuzumab therapy reported a 7.1% incidence of LVD, defined as a decrease in LVEF of 10% or more to an LVEF < 50, 1.7% incidence of symptomatic heart failure, and 0.5% incidence of severe heart failure, defined as New York Heart Association (NYHA) class III-IV symptoms [2]. Retrospective observational studies have reported a higher incidence of TIC ranging from 16 to 21% [5–9].

The mechanism of TIC is thought to be secondary to the temporary interruption of cardiomyocyte signalling pathways [10]. In contrast to anthracycline-induced cardiotoxicity, TIC may be reversible upon cessation of therapy [11]. An early study in a small cohort of patients with TIC showed that LVEF improved upon discontinuation of trastuzumab and introduction of LV enhancement therapy (LVETx), which included a combination of β blockers and angiotensin converting enzyme (ACE) inhibitors [12].

The reversibility of TIC highlights the importance of early cardiology consultation to improve both cardiac and oncologic outcomes through the institution of LVETx that may allow for continuation or early re-introduction of trastuzumab therapy. This is especially relevant in the context of emerging evidence suggesting that interruption of trastuzumab therapy may be associated with inferior oncologic outcomes [13].

With the emergence of the field of cardio-oncology and in the context of increasing demand for timely access to cardiology consultation in this unique population, dedicated cardio-oncology clinics have opened at centres across Canada, including the Vancouver General Hospital Cardio-Oncology Clinic, which opened in 2011. An important objective for cardio-oncology clinics, in general, is to enhance timely access to cardiology expertise for patients with cancer therapy-related cardiotoxicity and thus minimize treatment interruptions.

In this context, we designed a study aiming to describe the incidence of TIC in patients receiving adjuvant trastuzumab in the province of British Columbia (BC), explore the management strategies employed for these patients, and describe cardiovascular outcomes among patients experiencing cardiotoxicity over a 4 year period spanning the launch of a Cardio-Oncology Clinic.

## Methods

We conducted a retrospective cohort study of breast cancer patients in the province of BC treated with initial curative intent with Trastuzumab over a four-year period between January 1, 2010 and December 31, 2013. This period from 2010 to 2013 included the year prior to clinic opening, the year of the clinic’s soft opening, and the 2 years immediately after the cardio-oncology clinic opening in Vancouver. This clinic serves patients from all over the province both directly and by telehealth. The BC Cancer Agency’s institutional pharmacy database was utilized to identify patients who received at least one dose of Trastuzumab in BC during this time period. Patients were included in this study if they had a histologically confirmed diagnosis of HER2+ breast cancer, completed all of their therapy in BC, and were treated with initial curative intent. Patients with known metastases prior to trastuzumab initiation were excluded from our study. Patients were excluded if they did not have a baseline assessment of LV function and at least one subsequent measurement of LV function during trastuzumab therapy.

These charts were then audited for left ventricular function measurements to identify patients who experienced declines in left ventricular ejection fraction (LVEF) during trastuzumab therapy. The proportion of patients experiencing decline in LVEF was determined and reported descriptively. The degree of LVEF decline was also recorded.

Charts for patients who experienced a decline by greater than 10% to an absolute LVEF of < 50% were then reviewed more extensively for demographic information, breast cancer characteristics, cardiac comorbidities, anthracycline and trastuzumab dosing regimen, subsequent cardiac events breast cancer outcomes, cardiology referral, and heart failure management. Symptomatic left ventricular dysfunction was defined as having the presence of significant new onset dyspnea, orthopnea, paroxysmal nocturnal dyspnea, peripheral edema and/or pulmonary edema documented in clinical assessments. Minimal LVD was defined as LVEF nadir 45–49%, mild LVD was defined as LVEF nadir 40–44% and moderate-severe LVD defined as LVEF nadir < 40%. Completion of planned trastuzumab therapy was defined as receiving 17 cycles of trastuzumab, in keeping with local standard adjuvant treatment regimens. Treatment interruption was defined as delay of 6 weeks or more between trastuzumab doses.

It was not possible, using data available, to determine which patients were referred to the dedicated Cardio-Oncology Clinic vs. other cardiology practices. To approximate this in an exploratory analysis, patients treated in the health authority in which the clinic is located and referred to cardiology from 2011 onward were considered most likely to have been referred here, and were analysed as a “presumed cardio-oncology referral group”.

Baseline characteristics were compared between patients with differing severities of LVD, between those referred vs. not referred to cardiology, and between those completing vs. not completing trastuzumab using the Student’s t-test, Wilcoxon rank sum test, ANOVA, or Kruskall-Wallace ANOVA for continuous variables and the Chi square test or Fisher’s exact test for categorical variables. Continuous variables that were normally distributed were summarized as mean +/− standard deviation for normally distributed variables and with median values and interquartile ranges if not normally distributed. Continuous variables were tested for normality using the Shapiro-Wilk test. Paired t-tests or repeated measures ANOVA were used for comparisons involving changes in LVEF. Logistic regression was used to identify associations between patient characteristics and the outcomes of cardiology referral and completion of planned trastuzumab therapy, using variables felt a priori to be likely to influence these outcomes (including treatment setting, risk factors for cardiotoxicity, and markers of cancer risk). Mastectomy was used in regression models as a surrogate for tumor size and risk; sensitivity analyses were performed using T stage as an alternate marker of size and risk. Statistical analysis was performed using SAS 9.4 (SAS Institute Inc., Cary, NC).

## Results

### Patient characteristics

**Fig. 1:**
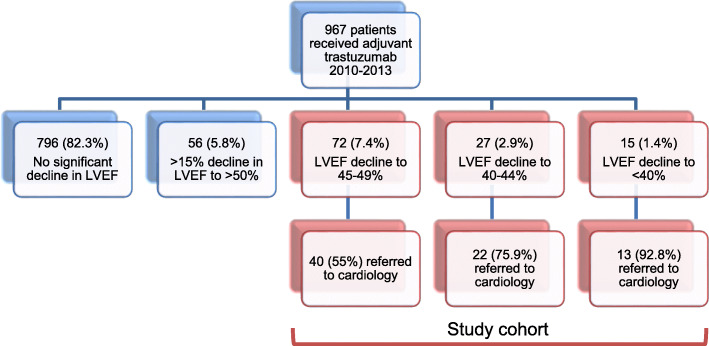
Flow chart of patients receiving adjuvant trastuzumab at BCCA from 2010 to 2013 based on the degree of left ventricular dysfunction and rate of referral to cardiology

We identified 967 patients with HER2+ breast cancer treated with adjuvant trastuzumab during the study period. Of these, 796 (82.3%) did not experience TIC and 114 (11.8%) patients experienced a decline of greater than 10% to a LVEF of < 50% during trastuzumab therapy and made up our study cohort (Fig. 1). The median number of LVEF measurements per patient was 8 (range 3–19). Six patients did not have further LVEF measurements after their initial decline and were therefore excluded from the analysis of LV recovery. LV function was measured using echocardiography or multiple gated acquisition (MUGA) scan in various hospital-based laboratories in British Columbia.

Our final cohort included 114 patients. Of these, 113 were female and one was male. The mean age was 56.6 ± 10.6 years. The median number of trastuzumab cycles received was 15 (IQR 8,17). One hundred one patients (89%) received anthracyclines with a median doxorubicin dose (or equivalent) of 240 mg/m^2^ (IQR 150,240). A history of cardiovascular disease was present in 5 patients (6%), and 57 patients (50%) had at least one cardiovascular risk factor (hypertension, dyslipidemia, diabetes, or cigarette smoking). The mean baseline LVEF of our cohort was 60.9%.

The lowest recorded LVEF value was 45–49% (“minimal LVD”) in 72 (63%) of patients, 40–44% (“mild LVD”) in 27 (24%), and < 40% (“moderate-severe LVD”) in 15 (13%). There were no significant differences in baseline clinical characteristics (including cardiovascular risk factors), cancer characteristics, or proportions of patients who underwent mastectomy or received radiation therapy between groups who developed minimal, mild and moderate-severe LVD (Table 1). Doses of anthracyclines were significantly higher among patients with moderate-severe LVD compared to other groups. Compared to those with minimal-mild LVD, patients who developed moderate-severe LVD were significantly more likely to be symptomatic.

**Table 1:** Characteristics and outcomes of study population by severity of LV dysfunction

	Moderate-severe (***n*** = 15)	Mild (***n*** = 27)	Minimal (***n*** = 72)	***P*** value
**Clinical characteristics**				
Age	55.7 ± 12.4	59.0 ± 9.6	55.9 ± 10.6	0.39
Post-menopausal	9 (60%)	22 (81%)	50 (69%)	0.40
Hypertension	4 (27%)	10 (37%)	25 (35%)	0.79
Diabetes	1 (7%)	2 (7%)	12 (17%)	0.42
Hyperlipidemia	1 (7%)	3 (11%)	9 (13%)	1.00
Cigarette smoking (current)	2 (13%)	8 (30%)	10 (14%)	0.19
Congestive heart failure	0	0	1 (1%)	1.00
Valve disease	0	0	1 (1%)	1.00
Arrhythmia	0	0	2 (3%)	1.00
Coronary artery disease	0	1 (4%)	1 (1%)	0.60
Stroke	0	0	0	N/A
HF symptoms with LVEF decline	8 (53%)	4(15%)	15 (21%)	**0.01**
**Cancer characteristics**				
Left-sided cancer	9 (60%)	14 (52%)	39 (54%)	0.10
Lymph node involvement	7 (47%)	14 (52%)	37 (51%)	0.94
**Treatment characteristics**				
Mastectomy	11 (73%)	24 (89%)	61 (85%)	0.42
Radiation	13 (87%)	21 (78%)	53 (74%)	0.55
Radiation dose (Gy)	55 (42.5,90)	80 (40,98.4)	53.75 (0,87.25)	0.45
Anthracycline	15 (100%)	25 (93%)	61 (85%)	0.23
Cumulative dose (mg/m2)	240 (240,240)	240 (150,240)	240 (150,240)	**0.04**
Trastuzumab cycles received	7 (4,11)	14 (10,17)	16 (10,17)	**0.002**
Trastuzumab interruption	15 (100%)	27 (100%)	67 (93%)	0.10
Trastuzumab re-started	6 (40%)	16 (59%)	43 (60%)	0.36
Completed planned trastuzumab	1 (7%)	10 (37%)	35 (49%)	**0.01**
**Other management**				
Cardiology referral	14 (93%)	21 (78%)	40 (56%)	**0.006**
Beta blocker	15 (100%)	14 (52%)	28 (39%)	**< 0.0001**
ACE inhibitor	15 (100%)	18 (67%)	38 (53%)	**0.002**
Loop diuretic	9 (60%)	7 (26%)	8 (11%)	**0.0001**
Mineralocorticoid receptor antagonist	9 (60%)	1 (4%)	1 (1%)	**< 0.0001**
**Outcomes**				
Breast cancer recurrence	3 (20%)	5 (19%)	12 (17%)	0.88
All cause mortality	1 (7%)	4 (15%)	6 (8%)	0.64

Values shown are counts (percentages), mean ± SD, or median (Q1,Q3). Abbreviations: *ACE* Angiotensin converting enzyme, *HF* Heart failure, *LVEF* Left ventricular ejection fraction

### Management and outcomes among patients referred to cardiology vs. not referred

Of 114 patients in our study cohort, 75 (66%) were referred to cardiology. In the entire study cohort, increasing severity of LVD was associated with significantly increased probability of being referred to cardiology (Table 1). Cardiology clinic referral rate ranged from 56% in patients with minimal LVD to 93% in patients with moderate-severe LVD. Referred patients were seen at a mean of 4.2 clinic visits over a median follow-up of 698 days.

Patients who were referred to cardiology were more likely to be prescribed LVETx and diuretics (Table 2). There were no significant differences in the proportions of patients taking any of these medications at baseline between those subsequently referred to cardiology and those who were not. Increasing severity of LVD was also associated with significantly increased probability of being prescribed β blockers, ACE inhibitors, mineralocorticoid receptor antagonists (MRA), and loop diuretics. All patients with moderate-severe LVD were started on both β blockers and ACE inhibitors, while β blockers and ACE inhibitors were started in 39 and 53%, respectively, of patients with minimal LVD.

**Table 2:** Management of patients who were or were not referred to cardiology

	Cardiology referral (*n* = 75)	No referral (*n* = 39)	*P* value
Medications			
β blockers	55 (73%)	2 (5%)	< 0.0001
ACE inhibitors	66 (88%)	5 (13%)	< 0.0001
MRA	10 (13%)	1 (3%)	0.06
Loop diuretic	21 (28%)	3 (8%)	0.01
Trastuzumab interruption	74 (99%)	35 (90%)	0.03
Trastuzumab re-started	40 (53%)	25 (64%)	0.27
Completed planned trastuzumab	26 (35%)	20 (51%)	0.09

Values shown are counts (percentages). Abreviations: *ACE* Angiotensin converting enzyme, *MRA* Mineralocorticoid receptor antagonist

After adjusting for severity of LV dysfunction and history of hypertension, patients referred to cardiology were more likely to be prescribed β blockers (adjusted OR 50.8, 95% CI 9.8–262.8), and ACE inhibitors (adjusted OR 132.8, 95% CI 16.5- > 999.99) compared with those who were not referred, but not more likely to be prescribed MRAs.

Although patients referred to a cardiologist had a slightly lower mean LVEF nadir (43.0% vs. 45.9%, *P* = 0.0005), the mean LVEF at last follow-up did not differ between the two groups (54.7% vs. 55.2%, *P* = 0.96). The mean LVEF change from nadir to last-follow-up also did not differ between the two groups (*P* = 0.073).

### Patterns of cardiology referral associated with launch of cardio-oncology clinic

**Fig. 2:**
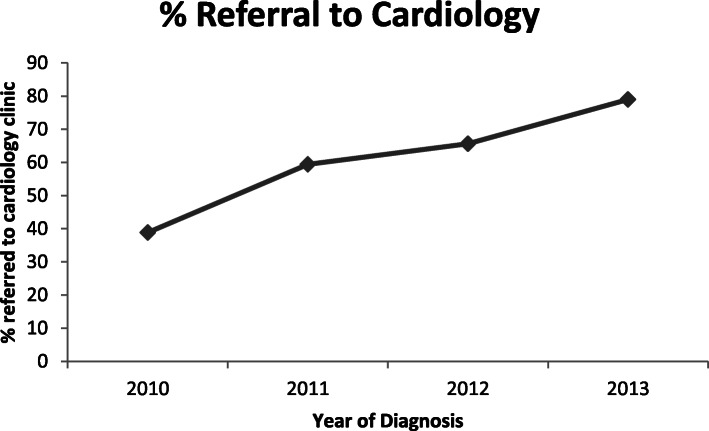
Temporal trend in cardiology referral rates for patients with LVEF drop to < 50%

**Fig. 3:**
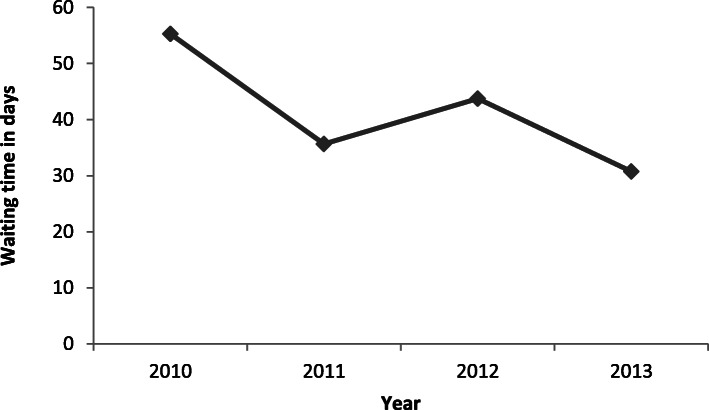
Temporal trend in waiting time from time of LVEF drop < 50% to cardiology consultation

The frequency of cardiology referral for LVD changed over the study period; of note, a cardio-oncology clinic opened in the quaternary care hospital associated with the cancer centre studied in 2011. In 2010, 45% of patients with treatment-emergent LVD were referred to a cardiologist for assessment. This proportion steadily rose to a peak of 80% by 2013 (adjusted *p* = 0.002 for change with time; Fig. 2). A non-significant decrease in time to cardiology consultation from the time of LVEF decline was observed, from 55.3 days in 2010 to 28.8 days in 2013 (*p* = 0.18, Fig. 3). Non-significant changes were also observed in the frequency of β blocker use (45% in 2010 to 81% in 2013) and ACE inhibitor use (73% in 2010 to 94% in 2013).

Patients in the “presumed cardio-oncology clinic referral” group had a significantly shorter time to cardiology consultation than those referred to other cardiology clinics (22.5 days vs. 38.0 days, *p* = 0.0098). Although this patient group was more likely to be prescribed β blockers (*p* = 0.048) and ACE inhibitors (*p* = 0.0049) compared to other patients overall, patients in the “presumed cardio-oncology clinic referral” group were not more likely to be prescribed β blockers, ACE inhibitors, or MRA compared to other patients who were referred to cardiology.

### Factors associated with cardiology referral

On univariable analysis, treatment centre (proximity to quaternary care centre vs. peripheral centre), non-mastectomy surgery (i.e. lumpectomy), lower LVEF at time of deterioration, and presence of symptoms were associated with increased probability of cardiology referral (Table 3). There were no significant differences in demographic characteristics, prevalence of baseline cardiovascular disease or risk factors, total number of trastuzumab cycles administered, anthracycline dose, or frequency of trastuzumab interruption between patients who were referred to cardiology and those who were not. On multivariable analysis, the strongest independent predictor of cardiology referral was the presence of symptoms (OR 8.0, 95% CI 1.5–42.9). Additional factors independently associated with cardiology referral included treatment in proximity to a quaternary care centre (OR 6.8, 95% CI 1.3–34.2) and later year of cancer diagnosis (OR 2.4 per year, 95% CI 1.4–4.3), while node positive disease (OR 0.18, 95% CI 0.06–0.56), mastectomy (OR 0.05, 95% CI 0.01–0.52), and LVEF nadir ≥45% (OR 0.14, 95% CI 0.05–0.46) were associated with a lower probability of referral. On sensitivity analysis, higher T stage was not associated with cardiology referral (OR 0.70, 95% CI 0.24–2.00).

**Table 3:** Characteristics of patients who were or were not referred to cardiology

	Cardiology referral (***n*** = 75)	No referral (***n*** = 39)	***P*** value
Year of diagnosis			0.076
2010	11 (15%)	9 (23%)	
2011	24 (32%)	15 (38%)	
2012	24 (32%)	11 (28%)	
2013	16 (21%)	4 (10%)	
Location			**0.041**
4^o^ care centre	20 (26%)	4 (10%)	
Peripheral centre	55 (73%)	35 (90%)	
Age at diagnosis (y)	57 ± 10	56 ± 12	0.714
T			0.877
0	1 (1%)	0	
1	27 (37%)	14 (36%)	
2	33 (45%)	20 (51%)	
3	3 (4%)	2 (5%)	
4	10 (14%)	3 (8%)	
N			0.143
0	40 (54%)	13 (34%)	
1	24 (32%)	19 (50%)	
2	6 (8%)	2 (5%)	
3	4 (5%)	4 (11%)	
Mastectomy	58 (77%)	38 (97%)	**0.005**
Radiation	58 (77%)	29 (74%)	0.723
Radiation dose (Gy)	55 (42.5,90)	80 (0,92.5)	0.667
Anthracycline dose (mg/m2)	240 (150,240)	240 (150,240)	0.298
Pre-existing CVD	4 (5%)	2 (5%)	0.963
CV risk factors	39 (52%)	18 (46%)	0.554
Baseline LVEF	61% ±6	60% ±7	0.435
LVEF at time of deterioration	45% (42,47)	47% (45,48)	**0.003**
Symptoms of HF	24 (32%)	3 (8%)	**0.004**

Values shown are counts (percentages), mean ± SD, or median (Q1, Q3). Abbreviations: *CV* Cardiovascular, *CVD* Cardiovascular disease, *HF* Heart failure, *LVEF* Left ventricular ejection fraction, *N* Nodal stage, *T* Tumour stage

### Management of Trastuzumab

The vast majority (94%) of patients in in the study cohort had an interruption in trastuzumab therapy at the time of decline in LVEF, with no difference between groups based on severity of LVD. More severe LVD was not associated with a lower probability of restarting trastuzumab but it was associated with a lower probability of receiving a complete course of trastuzumab and with receiving fewer cycles of trastuzumab overall. Overall, only 46 (40%) patients completed planned trastuzumab therapy, and only 26% of patients with LVEF nadir < 45% completed planned therapy.

On univariable analysis, treatment centre (proximity to quaternary care centre vs. peripheral centre), lymph node involvement, mastectomy, higher LVEF at time of deterioration, and higher LVEF nadir were associated with increased probability of receiving a full course of trastuzumab (Table 4). On multivariable analysis, an increased probability of receiving a full course of trastuzumab was independently associated with mastectomy (OR 5.1, 95% CI 1.1–23.0) and treatment in proximity to a quaternary care centre (OR 7.7, 95% CI 2.2–26.2), while moderate-severe LVD was associated with a reduced probability of receiving a full course (OR 0.07 vs. minimal LVD, 95% CI 0.01–0.74). Completion of trastuzumab was not significantly associated with referral to cardiology, age, or presence of heart failure symptoms. Sensitivity analysis was performed to test the assumption that mastectomy was a marker of larger tumor size. On multivariable analysis, a non-significant trend towards increased likelihood of receiving a full course was associated with T stage > 1 (OR 2.0, 95% CI 0.8–4.8).

**Table 4:** Characteristics of patients who did or did not receive full course of trastuzumab

	Full course received (***n*** = 46)	Incomplete course (***n*** = 68)	***P*** value
Year of diagnosis			0.725
2010	6 (13%)	14 (21%)	
2011	18 (39%)	21 (31%)	
2012	14 (30%)	21 (31%)	
2013	8 (17%)	12 (18%)	
Location			**0.003**
4^o^ care centre	16 (35%)	8 (12%)	
Peripheral centre	30 (65%)	60 (88%)	
Age at diagnosis	56 years ±11	57 years ±10	0.556
T			0.395
0	1 (2%)	0	
1	13 (28%)	28 (42%)	
2	23 (50%)	30 (45%)	
3	3 (7%)	2 (3%)	
4	6 (13%)	7 (10%)	
N			**0.0002**
0	14 (31%)	39 (58%)	
1	23 (51%)	20 (30%)	
2	3 (7%)	5 (7%)	
3	5 (11%)	3 (4%)	
Mastectomy	43 (93%)	53 (78%)	**0.026**
Radiation	37 (80%)	50 (74%)	0.395
Radiation dose (Gy)	80 (42.5,95)	52.5 (0,85.25)	0.074
Anthracycline dose	240 mg/m2 (240,240)	240 mg/m2 (150,240)	0.148
Pre-existing CVD	2 (4%)	4 (6%)	1.0
CV risk factors	21 (46%)	36 (53%)	0.445
Baseline LVEF	62% ±6	60% ±6	0.251
LVEF at time of deterioration	47% (45,48)	45% (42,47.5)	**0.005**
Symptoms of HF	7 (15%)	20 (30%)	0.080
Cardiology consult	26 (57%)	49 (72%)	0.086

Values shown are counts (percentages), mean ± SD, or median (Q1, Q3). Abbreviations: *CV* Cardiovascular, *CVD* Cardiovascular disease, *HF* Heart failure, *LVEF* Left ventricular ejection fraction, *N* Nodal stage, *T* Tumour stage

### Clinical outcomes

There were no significant differences in all-cause mortality, cardiac mortality, or cardiac events among patients with minimal, mild, and moderate-severe LVD. There were four cardiac events: two patients (LVEF nadir 30 and 35%) were admitted for decompensated CHF, one patient with LVEF nadir 48% was treated in the emergency department for symptomatic CHF, and one patient with LVEF nadir 39% had an acute coronary syndrome and underwent percutaneous revascularization.

**Fig. 4:**
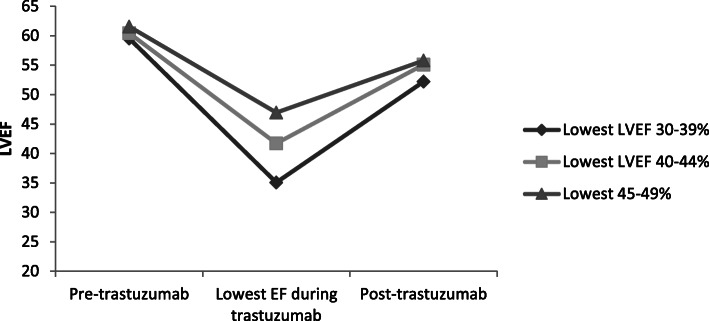
Assessment of left ventricular function pre- and post-trastuzumab therapy

For all patients with LVEF drop < 50%, the mean pre-treatment LVEF was 60.9% and mean post-treatment LVEF was 55.2%, with a mean decrease of 5.8% (*p* < 0.0001). At last follow up, the LVEF had recovered to > 50% in 98/108 (90.7%) of patients. No patients had a final LVEF < 45%. There were no differences in the probability of recovery between those who were referred to cardiology and those who were not, or between those with minimal LVD, mild LVD, or moderate-severe LVD (Fig. 4). Among patients who were started on both β blocker and ACE inhibitor, one of β blocker or ACE inhibitor, or neither, there was no statistical difference in the mean post-recovery LVEF between the three groups and all three groups had mean LVEF > 50% by the end of follow up.

## Discussion

In this retrospective study of patients receiving adjuvant trastuzumab therapy, we observed similar rates of TIC to those reported in previous observational studies. As expected, our data indicated that more severe LVD occurred in patients receiving higher doses of anthracyclines, and that patients with more severe LVD were more likely to develop symptomatic heart failure. In addition, our results provide a number of novel insights regarding the management of TIC in a contemporary real-world setting.

### Referral to cardiology

To our knowledge, our data are the first to describe temporal changes in real-world management strategies among patients with TIC and to demonstrate an association between access to a cardio-oncology clinic and these changing clinical care practices. We observed an increased cardiology referral rate, a decrease in wait time to cardiology assessment, and increased LVETx use over the study period, which spanned the time of establishment of a local dedicated cardio-oncology program. Cardio-oncology clinics may improve quality of care by improving ease of access to cardiology expertise and by educating community cardiologists around the nuances of treating this population [14].

The observed difference in wait time to cardiology assessment among patients treated in proximity to a cardio-oncology clinic (our “presumed cardio-oncology clinic referral” group) suggests an important benefit of specialized clinics in terms of ease of access, potentially reducing delays in cancer therapy. While we did not observe a difference in the use of LVETx between patients presumed to be referred to cardio-oncology and those referred to other cardiology clinics, the increase in use of these agents over time may indicate an increased awareness of cardio-oncology best practices among community cardiologists associated with the opening of the clinic. Furthermore, our observation that patients with TIC referred to any cardiologist were more likely to receive β blockers and ACE inhibitors compared with those who were not referred, even after adjusting for severity of LV dysfunction and hypertension, highlights the importance of facilitating rapid access to cardiology consultation; cardio-oncology clinics are often best able to achieve this.

To our knowledge, this is also the first study to examine the factors associated with cardiology referral in patients with TIC. As expected, severity of LVD and presence of heart failure symptoms influenced clinical decision making in patients with TIC. We had hypothesized that patients with pre-existing cardiovascular disease, cardiovascular risk factors, or more advanced age would also be more likely to be referred to cardiology. Surprisingly, none of these predictors of adverse cardiovascular outcomes was associated with referral. In fact, we observed that this decision was driven strongly by treatment setting (year and centre) and cancer characteristics. Our most surprising finding in this analysis was the negative association between high-risk cancer characteristics (node-positivity, mastectomy) and cardiology referral. Our data suggest that in the setting of high-risk breast cancer, oncologists may be less concerned with cardiotoxicity and prioritize completion of trastuzumab over cardioprotection.

### Use of LV enhancement therapy and clinical outcomes

While a large body of literature has described the incidence, risk factors, and outcomes of TIC in real-world populations [5–9], few studies have examined contemporary management strategies, particularly with respect to LVETx use. A previous study of patients with LVD during or after anthracycline or trastuzumab therapy at Stanford University Medical Center found that 42% of patients with asymptomatic cardiotoxicity and 89% of patients with symptomatic cardiotoxicity received a cardiology consultation [15]. These referral rates are similar to the referral rates observed in our cohort prior to the establishment of a cardio-oncology clinic. Similarly, the rates of LVETx use in our cohort were approximately equivalent to the Stanford cohort, in whom 40% of asymptomatic patients received LVETx.

In contrast to guidelines for management of heart failure patients in the general population, cardio-oncology guidelines [16–18] recommend LVETx with ACE inhibitors and β blockers for asymptomatic and mild LVD in the setting of cardiotoxic chemotherapy [17]. Extensive data support the use of these agents in patients with cardiotoxicity after anthracycline exposure [11, 19–23]. The evidence base for cardioprotection against TIC is less well established; while observational data have suggested that β blocker and ACE inhibitor use are associated with recovery of LV function in this population, other studies have demonstrated similar rates of recovery with the cessation of trastuzumab alone. Consistent with these latter studies, we did not observe an association between LV recovery and the use of LVETx. This may reflect the fact that trastuzumab therapy was interrupted in 93% of patients with TIC. Trastuzumab interruption alone may have been responsible for LV recovery in patients who did not receive ACE inhibitors or β blockers. However, as all patients with severe LV dysfunction were started on ACE inhibitor and β blocker treatment, we are unable to assess if trastuzumab treatment interruption alone would have been sufficient for LV recovery.

### Completion of Trastuzumab therapy

A concerning proportion of patients in our study did not complete planned trastuzumab therapy. We found that completion of trastuzumab therapy was associated with both oncologic and cardiac factors, including markers of advanced cancer and severity of LVD. Interruptions in trastuzumab may be associated with worse cancer outcomes [13], and it is therefore intuitive that oncologists would be more likely to fully treat patients with markers of higher risk cancer. Similarly, we were not surprised that trastuzumab was less likely to be completed in patients with greater degrees of LVD.

An important emerging question in cardio-oncology is whether it is safe to continue trastuzumab in patients with mild asymptomatic treatment-emergent LVD, using cardioprotective medications to support cardiac function through therapy, with the goal of delivering optimal cancer therapy while minimizing cardiac risk [24]. Early data suggest that aggressive use of β blockers and ACE inhibitors may allow safe continuation of trastuzumab therapy in breast cancer patients with treatment-emergent mild LVD [25]. Larger studies are needed to determine the safety and efficacy of this approach, but if confirmed, these findings suggest that the greatest benefit of cardiology referral may be among patients with high-risk cancers to enable optimal oncologic therapy while supporting cardiac function. This approach would depend on access to cardiology expertise and the ability to rapidly up-titrate cardioprotective medications. Consistent with this, patients referred to cardiology in our study were not less likely to complete their planned trastuzumab therapy and were more likely to receive LVETx.

### Strengths and limitations

Strengths of our study include the detailed chart review used for data collection in a field where many studies rely on observational data, as well our access to charts for over 85% of breast cancer patients treated in our province through a provincial Cancer Agency electronic medical record. There are also limitations to our study. By relying on chart review, we may have missed cardiovascular events, medications, and visits that occurred outside the Cancer Agency and that were not subsequently documented in charts. Our follow up was limited to a median of 2 years duration and did not capture long term cardiovascular complications. However, most cardiovascular events related to trastuzumab therapy occur during or soon after therapy and would likely be captured in our cardio-oncology clinic visit consultations [26]. The changes in cardiovascular referral patterns and medication use may simply reflect temporal trends as opposed to being a result of access to a cardio-oncology clinic. Contemporary cardio-oncology practice has evolved over the years and with increased use of cardioprotective medications, the treatment interruptions reported in this study may have changed over time.

We utilized a “presumed cardio-oncology clinic referral” cohort in our analysis, but while we are confident that there was broad use of the clinic among referring breast oncologists from the time of clinic launch, we cannot be certain that all of these patients were referred to the cardio-oncology clinic as opposed to other local cardiologists. The short time to consultation does, however, suggest that a majority of patients have been accurately classified. We used mastectomy as a surrogate for tumor size and cancer risk; using T stage as an alternative did not lead to statistically significant associations. Mastectomy status may therefore reflect a more complex risk analysis than simply tumor size (perhaps incorporating patient preference, physician practice patterns, other markers of tumor behavior, and/or surgical risk). Nonetheless, it was a consistent predictor of management decisions in our cohort. Finally, our small sample size and retrospective methodology preclude us from making conclusions regarding the efficacy of LVETx.

## Conclusions

In conclusion, we observed a steady increase in the rate of cardiology referral and use of LV enhancement therapies in breast cancer patients with adjuvant trastuzumab-induced cardiotoxicity over a period spanning the launch of a provincial cardio-oncology clinic. We identified opportunities for improvement in the management of this population, while observing that the presence of a cardio-oncology clinic was associated with greater likelihood of referral to a cardiologist and improved adherence to guideline-based management among patients with TIC. Decisions to complete trastuzumab therapy and to refer patients to cardiology were associated with treatment setting, cardiovascular factors, and cancer-related factors. Patients with higher-risk breast cancer may be less likely to be referred to cardiology. This identifies an important care gap, as referral to cardiology and timely initiation of cardioprotective therapies may offer opportunities for more complete cancer therapy.

## Data Availability

The data that support the findings of this study are available from the corresponding author but restrictions apply to the availability of these data, which were used with ethics approval from the BC Cancer Agency for the current study, and so are not publicly available. Data are however available from the authors upon reasonable request and with permission of the UBC CREB.

## Abbreviations

ACE: Angiotensin converting enzyme
BC: British Columbia
HER2: Human epidermal growth factor receptor-2
LV: Left ventricular
LVD: Left ventricular dysfunction
LVEF: Left ventricular ejection fraction
LVETx: Left ventricular enhancement therapy
NYHA: New York Heart Association
TIC: Trastuzumab-induced cardiotoxicity

## References

[1] Hudis CA (2007). Trastuzumab — mechanism of action and use in clinical practice. New Engl J Med.

[2] Piccart-Gebhart M, Procter M, Leyland-Jones B, Goldhirsch A, Untch M, Al E (2005). Trastuzumab after adjuvant chemotherapy in HER2-positive breast cancer. N Engl J Med.

[3] Slamon D, Eiermann W, Robert N (2011). Adjuvant Trastuzumab in HER2-positive breast cancer. N Engl J Med.

[4] Romond EH, Perez EA, Bryant J, Suman VJ, Geyer CE, Davidson NE, Tan-Chiu E, Martino S, Paik S, Kaufman PA, Swain SM, Pisansky TM, Fehrenbacher L, Kutteh LA, Vogel VG, Visscher DW, Yothers G, Jenkins RB, Brown AM, Dakhil SR, Mamounas EP, Lingle WL, Klein PM, Ingle JN, Wolmark N (2005). Trastuzumab plus adjuvant chemotherapy for operable HER2-positive breast Cancer. N Engl J Med.

[5] Tang GH, Acuna SA, Sevick L, Yan AT, Brezden-Masley C (2017). Incidence and identification of risk factors for trastuzumab-induced cardiotoxicity in breast cancer patients: an audit of a single “real-world” setting. Med Oncol.

[6] Yu AF, Yadav NU, Eaton AA, Lung BY, Thaler HT, Liu JE, Hudis CA, Dang CT, Steingart RM (2015). Continuous Trastuzumab therapy in breast Cancer patients with asymptomatic left ventricular dysfunction. Oncologist..

[7] Matos E, Jug B, Blagus R, Zakotnik B. A prospective cohort study on Cardiotoxicity of adjuvant Trastuzumab therapy in breast Cancer patients. Arq Bras Cardiol. 2016:40–7. 10.5935/abc.20160084. 10.5935/abc.20160084 PMC4976955 27305108

[8] Gunaldi M, Duman BB, Afsar CU, Paydas S, Erkisi M, Kara IO, Sahin B (2016). Risk factors for developing cardiotoxicity of trastuzumab in breast cancer patients: an observational single-Centre study. J Oncol Pharm Pract.

[9] Sawaya H, Sebag IA, Plana JC, Januzzi JL, Ky B, Cohen V, Gosavi S, Carver JR, Wiegers SE, Martin RP, Picard MH, Gerszten RE, Halpern EF, Passeri J, Kuter I, Scherrer-Crosbie M (2011). Early detection and prediction of cardiotoxicity in chemotherapy-treated patients. Am J Cardiol.

[10] Ewer MS, Ewer SM (2010). Cardiotoxicity of anticancer treatments: what the cardiologist needs to know. Nat Rev Cardiol.

[11] Seicean S, Seicean A, Plana JC, Budd GT, Marwick TH (2012). Effect of statin therapy on the risk for incident heart failure in patients with breast cancer receiving anthracycline chemotherapy: an observational clinical cohort study. J Am Coll Cardiol.

[12] Ewer MS, Vooletich MT, Durand JB, Woods ML, Davis JR, Valero V, Lenihan DJ (2005). Reversibility of trastuzumab-related cardiotoxicity: new insights based on clinical course and response to medical treatment. J Clin Oncol.

[13] Gibson J, Yao RJ, Davis M, Simmons C (2017). The impact of mild left ventricular dysfunction on trastuzumab use and oncologic outcomes in early stage breast cancer therapy. J Clin Oncol.

[14] Barac A, Murtagh G, Carver JR, Chen MH, Freeman AM, Herrmann J, Iliescu C, Ky B, Mayer EL, Okwuosa TM, Plana JC, Ryan TD, Rzeszut AK, Douglas PS (2015). Cardiovascular health of patients with cancer and cancer survivors: a roadmap to the next level. J Am Coll Cardiol.

[15] Yoon GJ, Telli ML, Kao DP, Matsuda KY, Carlson RW, Witteles RM (2010). Left ventricular dysfunction in patients receiving cardiotoxic cancer therapies: are clinicians responding optimally?. J Am Coll Cardiol.

[16] Hamo CE, Bloom MW, Cardinale D, Ky B, Nohria A, Baer L, Skopicki H, Lenihan DJ, Gheorghiade M, Lyon AR, Butler J (2016). Cancer therapy-related cardiac dysfunction and heart failure: part 2: prevention, treatment, guidelines, and future directions. Circ Heart Fail.

[17] Virani SA, Dent S, Brezden-Masley C, Clarke B, Davis MK, Jassal DS, Johnson C, Lemieux J, Paterson I, Sebag IA, Simmons C, Sulpher J, Thain K, Thavendiranathan P, Wentzell JR, Wurtele N, Côté MA, Fine NM, Haddad H, Hayley BD, Hopkins S, Joy AA, Rayson D, Stadnick E, Straatman L (2016). Canadian cardiovascular society guidelines for evaluation and Management of Cardiovascular Complications of Cancer therapy. Can J Cardiol.

[18] Zamorano JL, Lancellotti P, Rodriguez Muñoz D, Aboyans V, Asteggiano R, Galderisi M, Habib G, Lenihan DJ, Lip GYH, Lyon AR, Lopez Fernandez T, Mohty D, Piepoli MF, Tamargo J, Torbicki A, Suter TM (2016). 2016 European Society of Cardiology position paper on cancer treatments and cardiovascular toxicity. Eur Heart J.

[19] Bosch X, Rovira M, Sitges M, Domènech A, Ortiz-Pérez JT, de Caralt TM, Morales-Ruiz M, Perea RJ, Monzó M, Esteve J (2013). Enalapril and carvedilol for preventing chemotherapy-induced left ventricular systolic dysfunction in patients with malignant hemopathies: the OVERCOME trial (prevention of left ventricular dysfunction with enalapril and caRvedilol in patients submitted t). J Am Coll Cardiol.

[20] Gulati G, Heck SL, Ree AH, Hoffmann P, Schulz-Menger J, Fagerland MW, Gravdehaug B, von Knobelsdorff-Brenkenhoff F, Bratland Å, Storås TH, Hagve TA, Røsjø H, Steine K, Geisler J, Omland T (2016). Prevention of cardiac dysfunction during adjuvant breast cancer therapy (PRADA): a 2 × 2 factorial, randomized, placebo-controlled, double-blind clinical trial of candesartan and metoprolol. Eur Heart J.

[21] Seicean S, Seicean A, Alan N, Plana JC, Budd GT, Marwick TH (2013). Cardioprotective effect of -Adrenoceptor blockade in patients with breast Cancer undergoing chemotherapy: follow-up study of heart failure. Circ Heart Fail.

[22] Pituskin E, Mackey JR, Koshman S, Jassal D, Pitz M, Haykowsky MJ, Pagano JJ, Chow K, Thompson RB, Vos LJ, Ghosh S, Oudit GY, Ezekowitz JA, Paterson DI (2017). Multidisciplinary approach to novel therapies in cardio-oncology research (MANTICORE 101-breast): a randomized trial for the prevention of trastuzumab-associated cardiotoxicity. J Clin Oncol.

[23] Guglin M, Krischer J, Tamura R (2019). Randomized Trial of Lisinopril Versus Carvedilol to Prevent Trastuzumab Cardiotoxicity in Patients With Breast Cancer. Randomized Controlled Trial.

[24] Jones AL, Barlow M, Barrett-Lee PJ, Canney PA, Gilmour IM, Robb SDPC (2009). Management of cardiac health in trastuzumab-treated patients with breast cancer : updated United Kingdom National Cancer Research Institute recommendations for monitoring. Br J Cancer.

[25] Leong D, Cosman T, Albussein M, Tyagi NKKS (2019). Safety of continuing Trastuzumab despite mild Cardiotoxicity: a phase I trial. JACC Cardiooncol.

[26] Goldhar HA, Yan AT, Ko DT, Earle CC, Tomlinson GA, Trudeau ME, Krahn MD, Krzyzanowska MK, Pal RS, Brezden-Masley C, Gavura S, Lien K, Chan KKW (2016). The temporal risk of heart failure associated with adjuvant trastuzumab in breast cancer patients: a population study. J Natl Cancer Inst.

